# Surgical treatment of a calcified, amorphous tumor of the right ventricle complicated with thrombosis of the right pulmonary artery in an adult male: a case report

**DOI:** 10.1186/s13256-016-0873-z

**Published:** 2016-04-12

**Authors:** Efrosina Kajo, Edvin Prifti, Aurora Knuti, Arben Baboci, Merita Zeka

**Affiliations:** Department of Cardiac Surgery, University Hospital Center, Tirane, Albania

**Keywords:** Calcification, Cardiac tumor, Pulmonary emboli, Tricuspid and pulmonary valves

## Abstract

**Background:**

A calcified amorphous tumor of the heart is an extremely rare cardiac mass.

**Case presentation:**

A 32-year-old Albanian man presented to our hospital with fatigue, shortness of breath, progressive dyspnea, and right congestive heart failure. Echocardiography and chest computed tomography revealed a giant, calcified right ventricular mass that originated between the papillary muscles and the trabeculae and extended to the pulmonary valve. The patient underwent surgery with excision of the mass, replacement of the pulmonary valve with a biological one, and repair of the tricuspid valve. His histopathological examination revealed that the mass was a calcified, amorphous tumor. His postoperative course was uneventful.

**Conclusions:**

The clinical presentation of the calcified amorphous tumor is similar to that of other cardiac tumors, so surgical excision is mandatory. Histopathological examination remains the gold standard for an accurate diagnosis.

## Background

A calcified amorphous tumor of the heart (CAT) is a rare, nonneoplastic cardiac tumor or intracavitary cardiac mass composed of calcium deposits in a background of amorphous, degenerating, fibrinous material. This cardiac tumor may be confused with primary cardiac neoplasms such as a calcified cardiac myxoma or calcified thrombi. Only a few cases of this rare lesion have been reported in the literature [1–4]. Histopathological examination is the most exact method for making the diagnosis.

## Case presentation

**Fig. 1:**
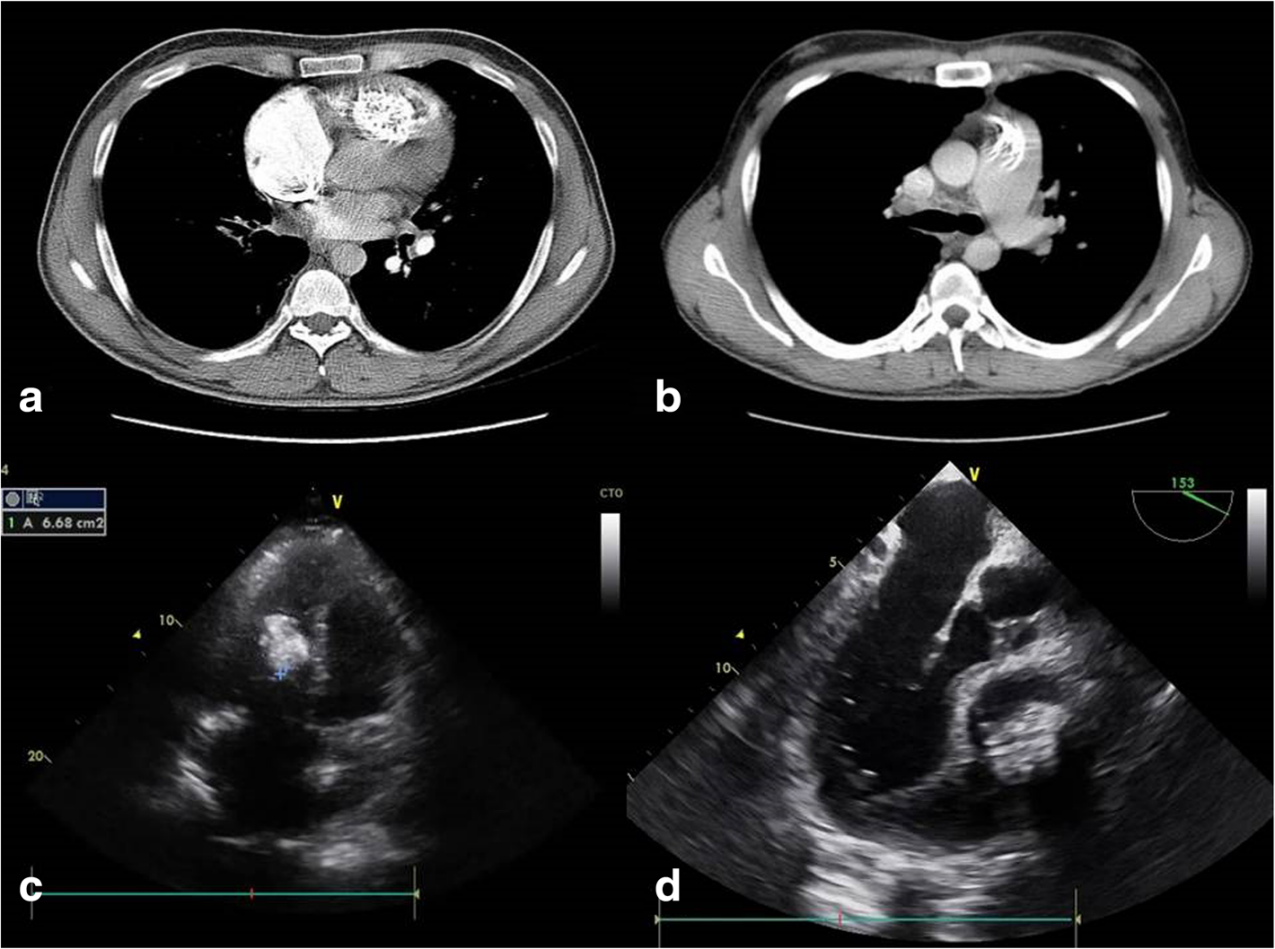
**a** Chest computed tomographic scan demonstrates an approximately 4 × 10–cm calcified mass in the right ventricle attached to the pulmonary valve. **b** Thrombosis of the origin of the right pulmonary artery is visualized with uniform contrast enhancement of the pulmonary trunk and the left pulmonary artery. **c** Transthoracic echocardiographic study and **d** transesophageal echocardiographic scan reveal a calcified mass in the right ventricle attached to the pulmonary valve

A 32-year-old Albanian man presented to our hospital with fatigue, cough, shortness of breath and progressive dyspnea, liver distention, and edema in the lower extremities, suggestive of right congestive heart failure. He was first treated for tracheobronchitis. Contrast-enhanced chest computed tomography (CT) was performed, which revealed a large, calcified right ventricular mass measuring 4 × 10 cm (Fig. 1a), a dilated pulmonary trunk approximately 4 cm in diameter, and thrombosis at the origin of the right pulmonary artery. The pulmonary trunk and left pulmonary branch were visualized with uniform contrast enhancement (Fig. 1b).

The patient had a family history of antithrombin III (AT3) deficiency. He had mild AT3 deficiency at 60 U/ml (normal AT3 value is 80 U/ml). His two sisters had pulmonary thromboembolisms and were being treated with major anticoagulants. The patient’s parathyroid hormone, phosphorus, and serum calcium concentrations were normal. The results of his routine laboratory examinations were within normal limits, except for a high level of bilirubin due to biliary stasis. He had no thrombosis of the venous circulation in his legs.

Transthoracic echocardiography (TTE) (Fig. 1c) and transesophageal echocardiography (TEE) (Fig. 1d) were performed. The imaging studies showed a 4 × 7–cm right ventricular mass that was hyperechogenic and calcified, and it appeared to be broad-based in the papillary muscles and trabeculae of the right ventricle with extension into the infundibulum and pulmonary valve. Slight tricuspid annular calcification was also recognized. The mass moved with ventricular wall contraction; no prolapse through the tricuspid valve into the right atrium was observed; and the septal tricuspid valve was hyperechogenic. The right atrium and right ventricle were dilated with volume and pressure overload. The patient’s pulmonary pressure was high, up to 70 mmHg.

**Fig. 2:**
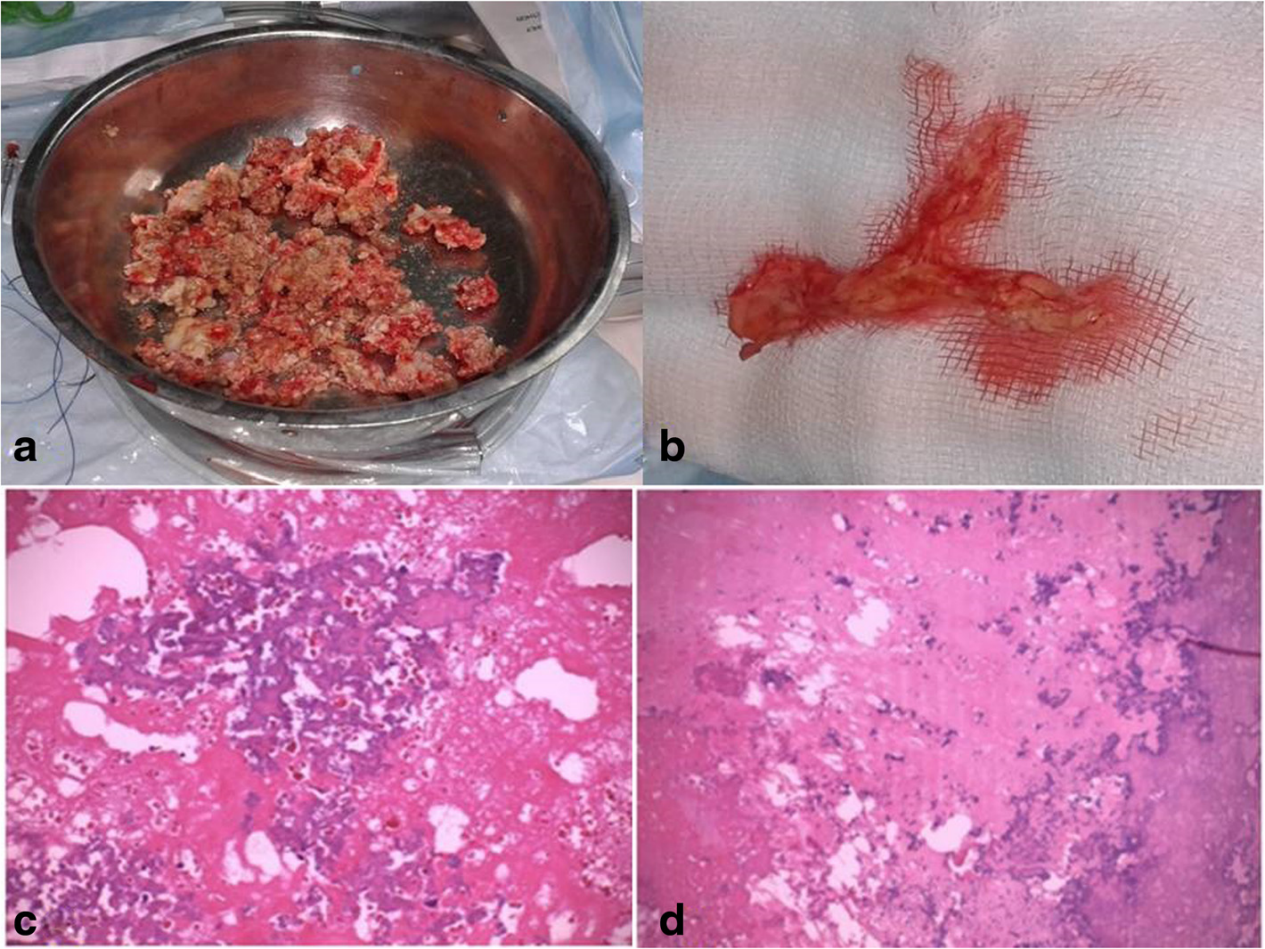
**a** Material remnants of the calcified mass removed from the right ventricle with surgical intervention. The remnants were conglomerated and had high consistency. **b** On the right side, the material of the pulmonary artery with a fibrinoid aspect and calcium nodes is seen. **c** Histopathological examination revealed a homogeneous, eosinophilic, largely acellular lesion with extensive areas of dystrophic calcification (hematoxylin and eosin stain, original magnification ×10). **d** The amorphous, fibrin-like material and hyaline formation in some areas is mixed with extravasated erythrocytes, spumous cells, and mild inflammatory cells

After cardiac CT and TEE were performed, the patient underwent surgical resection of the mass with a clinical diagnosis of calcified thrombi or any other cardiac neoplasms. Surgical intervention on 2 December 2014 consisted of right ventriculotomy, which revealed a giant calcified mass from the apex to the medial pulmonary artery (Fig. 2a and b). The pulmonary valve was damaged due to the CAT and was practically nonexistent. Substitution of the pulmonary valve with a Freestyle stentless bioprosthesis (number 25; Medtronic, Minneapolis, MN, USA), repair of the tricuspid valve (cleft suture), and extensive endarterectomy of right pulmonary branch were performed. The specimen was sent for histopathological examination.

Macroscopically, the material extracted from the right ventricle was of a conglomerated, reddish-brown, dry, clotlike consistency with a tendency to crumble when cut, and it was calcified and had high consistency (Fig. 2a and b). Focally mineralized calcification was present in the center of the tumor. Decalcification was performed before the tumor was processed for histological examination. Microscopically, the sections showed a lesion composed of a background of eosinophilic, amorphous material, possibly degenerated fibrin, with areas of dense calcification and focal chronic inflammation and aggregates of spumoseus histiocytes (Fig. 2c and d). The material of the pulmonary artery had a fibrinoid aspect and calcium nodes. No mitoses or malignant elements were seen.

**Fig. 3:**
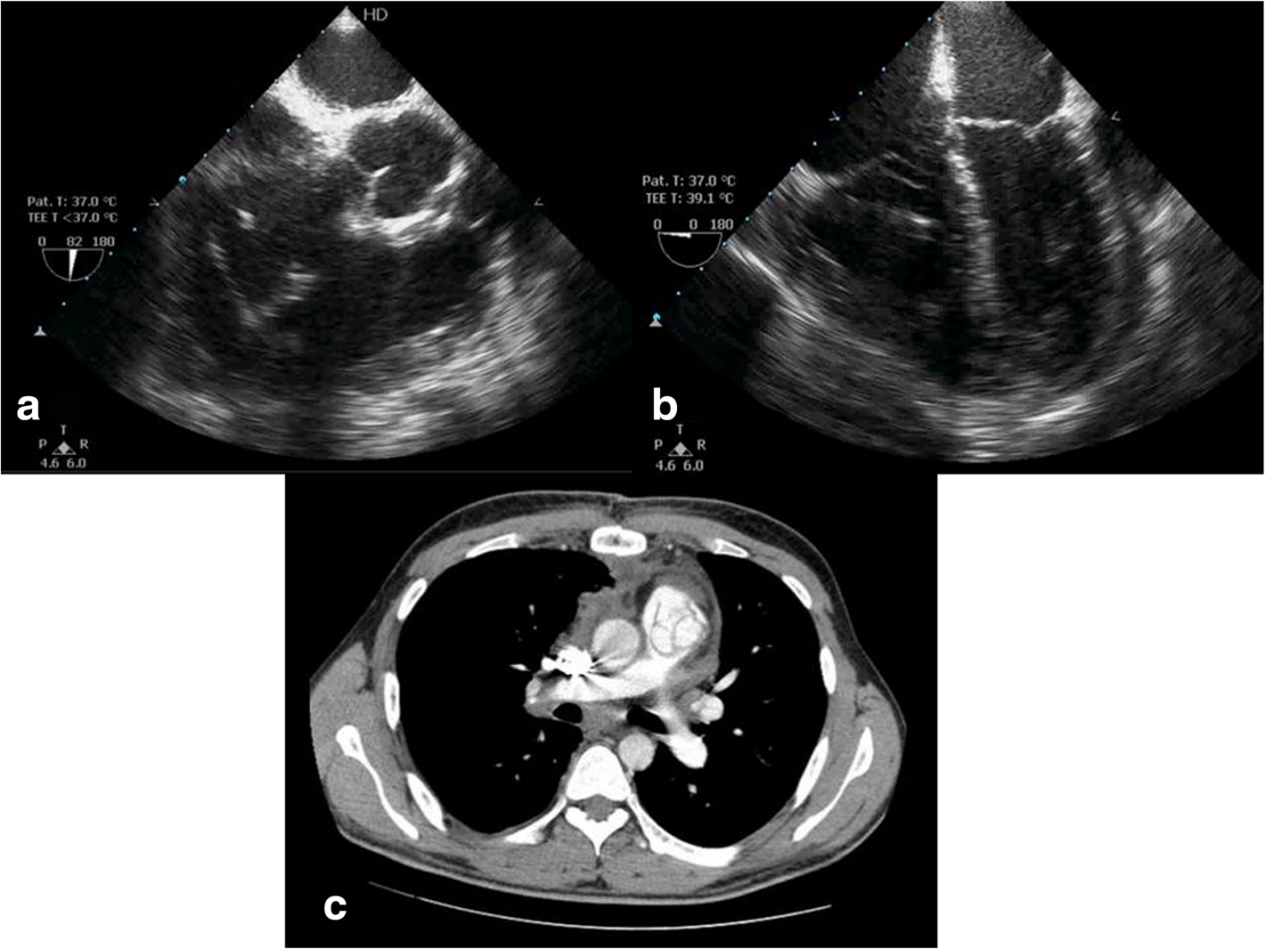
**a** and **b** Postoperative transesophageal echocardiography demonstrates no cardiac mass, the biologic prosthesis in pulmonary position, free tricuspid valve, and dilation of the right ventricle. **c** Postoperative chest computed tomography demonstrates good contrast visualization of the trunk and the two branches of the pulmonary artery

Considering the clinical and histological features, a diagnosis of CAT was made. The patient’s postoperative period was uneventful. Postoperative TEE was performed (Fig. 3a and b). No cardiac mass was seen; the function of the pulmonary prosthesis was normal; the patient’s maximal gradient was 25 mmHg; no pulmonary regurgitation was present; the infundibulum was free of mass; no volume or pressure overload of the right ventricle was present; mild tricuspid regurgitation was present; and the patient’s pulmonary pressure was 35 mmHg.

Postoperative contrast-enhanced chest CT showed no cardiac mass (Fig. 3c). The image provided good contrast visualization of the free right branch of the pulmonary artery that had been occluded before surgery. The patient was successfully treated and discharged from the hospital. Two months after his operation, the patient was healthy. He has to be followed with echocardiography for any possible future recurrence.

## Discussion

Primary cardiac tumors are rare, and the great majority of them are benign neoplasms [2]. The most common benign cardiac tumors are myxomas, accounting for 50 % of all benign cardiac tumors; however, not all cardiac masses are neoplasms. For instance, intramural thrombi are great mimics of neoplasms [3, 5]. Regardless of the nature of a cardiac mass (neoplastic or not), excision of the lesion is important due to the potential danger of obstruction or embolization [5–8].

CAT is a rare entity that was first described by Reynolds *et al*. in 1997 [3]. A review of the literature of 16 previously reported cases is also included [1, 3]. The rarity of this lesion is borne out by the fact that a 29-year review done at the Mayo Clinic yielded only 11 such cases [3]. On the basis of the findings in the Mayo Clinic series, CAT can originate in any cardiac chamber, tumor size and configuration are variable, and most of the tumors are located in intracavitary spaces and are motionless. They are described as nonneoplastic intracardiac lesions composed of nodules of calcium on a background of amorphous, fibrinous material with degeneration and focal chronic inflammation [3, 8].

Histologically, CAT consists of calcification and eosinophilic amorphous material in a background of dense, collagenous, fibrous tissue [2]. The mean age at the time of diagnosis is about 52 years (with an age range from 16 to 75 years) [1, 2, 9]. There is a slight female predominance; however, our patient was a 32-year-old man.

The clinical presentation of CAT is similar to that of other cardiac masses (that is, dyspnea, syncope, or symptoms related to embolism). Differential clinical diagnoses include cardiac calcified myxoma or fibroma, calcified cardiac tuberculoma, thrombi, emboli, vegetations, and intracardiac carcinosis, especially in patients with hemodialyzed end-stage renal disease and abnormal calcium and phosphorus metabolism; other benign and malignant cardiac tumors may be included as well [10–12].

TTE, TEE, and other examinations help identify the location, echogenicity, and morphology of cardiac masses. CATs are described as pedunculated, diffusely calcified masses visualized on echocardiograms [13–15]. They can grow in any cardiac chamber and on the mitral valve as well, but they are predominantly found on the left ventricle. In our patient, the CAT was found on the right ventricle, chordae tendineae, papillary muscles, and the infundibulum, reaching the pulmonary valve. These masses can reach a size of up to 9 cm in the greatest dimension; our patient’s tumor measured 7 cm.

In the absence of distinctive clinical and imaging features, a preoperative differentiation between neoplastic and nonneoplastic lesions remains difficult. Surgical resection of CATs is prompted by clinical suspicion of malignancy or due to obstructive or embolic complications [2, 4, 6, 13, 15]. Our patient presented with a right branch pulmonary embolism.

Hence, histological diagnosis is the gold standard for a definitive diagnosis [2, 4, 9, 16]. In our patient, extensive sampling failed to reveal any myxomatous tissue characteristic of myxomas. Thrombi may undergo mummification and calcification and mimic CAT. The absence of predisposing conditions for thrombosis and infrequent presence of hemosiderin differentiate CAT from an organizing thrombus [3, 17]. Although the pathogenesis of CAT is uncertain, association with organized thrombi, primary or secondary hypercoagulability [16], or abnormal calcium and phosphorus metabolism, especially in hemodialyzed patients [10–12], has been suggested.

Surgical excision is mandatory for diagnosis and treatment [2, 4, 6]. The majority of patients reported have had a benign course after surgical excision. Sometimes, some residual calcium may be seen [3]. Postoperative recurrence of CAT has rarely been reported [1, 16, 18]. TEE can reveal residual tumor. Regular postoperative follow-up with cardiac imaging studies is recommended, especially in cases of incomplete resection [9, 14, 16, 17].

## Conclusions

CAT is a rare intracardiac nonneoplastic mass. Its clinical presentation is similar to that of other cardiac tumors, so surgical excision is mandatory. Histopathological examination remains the gold standard for an accurate diagnosis. Regular postoperative follow-up with cardiac imaging studies is recommended to detect any possible recurrence.

## Consent

Written informed consent was obtained from the patient for publication of this case report and any accompanying images. A copy of the written consent is available for review by the Editor-in-Chief of this journal.

## Abbreviations

AT3: antithrombin III
CAT: calcified amorphous tumor of the heart
CT: computed tomography
TEE: transesophageal echocardiography
TTE: transthoracic echocardiography
